# Subventricular zone involvement is associated with worse outcome in glioma WHO grade 2 depending on molecular markers

**DOI:** 10.1038/s41598-021-97714-5

**Published:** 2021-10-08

**Authors:** Philipp Karschnia, Jonathan Weller, Jens Blobner, Veit M. Stoecklein, Mario M. Dorostkar, Kai Rejeski, Robert Forbrig, Maximilian Niyazi, Louisa von Baumgarten, Jorg Dietrich, Joerg-Christian Tonn, Niklas Thon

**Affiliations:** 1 Department of Neurosurgery, Ludwig Maximilians University, Marchioninistrasse 15, 81377 Munich, Germany; 2 German Cancer Consortium (DKTK), Partner Site Munich, Munich, Germany; 3 Department of Neurology, Massachusetts General Hospital Cancer Center, Harvard Medical School, Boston, MA USA; 4 Center for Neuropathology and Prion Research, Ludwig-Maximilians-University, Munich, Germany; 5 Department of Medicine III, Ludwig-Maximilians-University, Munich, Germany; 6 Department of Neuroradiology, Ludwig-Maximilians-University, Munich, Germany; 7 Department of Radiation Oncology, Ludwig-Maximilians-University, Munich, Germany; 8 Department of Neurology, Ludwig-Maximilians-University, Munich, Germany

**Keywords:** CNS cancer, Predictive markers, Prognostic markers, CNS cancer, Dementia, Chemotherapy, Radiotherapy, Tumour biomarkers, Surgical oncology

## Abstract

Neural stem cells within the subventricular zone were identified as cells of origin driving growth of high-grade gliomas, and anatomical involvement of the subventricular zone has been associated with an inferior clinical outcome. Whether the association between poor outcome and subventricular zone involvement also applies to glioma of lower grades is unclear. We therefore analysed a retrospective cohort of 182 patients with glioma grade 2 (according to the WHO 2016 classification) including 78 individuals (43%) with subventricular zone involvement. Patients with and without subventricular zone involvement did not differ in regard to demographics, histopathology, and molecular markers. Notably, subventricular zone involvement was a negative prognostic marker for malignant progression and overall survival on uni- and multivariate analysis. When patients were stratified according to the cIMPACT-NOW update 6, subventricular zone involvement was negatively associated with outcome in IDH-wildtype astrocytomas and 1p19q-codeleted oligodendrogliomas but not in IDH-mutant astrocytomas. Collectively, subventricular zone involvement may represent a risk factor for worse outcome in glioma WHO grade 2 depending on the molecular tumor signature. The present data confirm the relevance of molecular glioma classifications as proposed by the cIMPACT-NOW update 6. These findings warrant evaluation in prospective cohorts.

## Introduction

Gliomas WHO grade 2 are a distinct subgroup of primary central nervous system (CNS) neoplasms arising from the supporting glial cells of the cerebral parenchyma. Such tumors account for 5% of all primary brain tumors^1^. Although gliomas WHO grade 2 as a group are associated with a prolonged natural history and favourable prognosis when compared to high-grade gliomas, the vast majority are life-limiting. Median survival is 13 years when an early combination of surgical and medical treatment is administered^2^. However, several studies have advocated for less aggressive therapeutic approaches, and so far no definitive 'standard of care' has been established^3,4^. Molecular markers prognostic of outcome have been introduced to guide therapy^5^.

The subventricular zone of the lateral ventricle may represent the largest niche of neural stem cells in the adult human brain^6^. Neural stem cells within the subventricular zone have been found to substantially drive growth of high-grade glioma^7^, and closer proximity of high-grade glioma to the subventricular zone is correlated with a genetic stem cell signature^8^. Consequently, involvement of the subventricular zone appears to be associated with decreased survival in high-grade glioma^9^. Whether involvement of the subventricular zone also translates into clinical differences in glioma WHO grade 2 remains to be elucidated. Such an association might be extrapolated from previous studies, however, these studies did not account for prognostic molecular markers which may have confounded analysis^10,11^. The recent cIMPACT-NOW update 6 has introduced new tumor entities formerly summarized as WHO grade 2 based on such molecular markers^12,13^. Therapeutic implications of subventricular zone involvement in glioma WHO grade 2 constitute another area of uncertainty.

In the present study, we describe a large cohort of 182 adult patients with histologically verified glioma WHO grade 2 treated at a single academic neuro-oncology centre. Based on this cohort, we outline the institutional experience for incidence, management, and outcome of patients with subventricular zone involvement.

## Materials and methods

### Study population

This study was approved by the Institutional Review Board of the Ludwig Maximilians University in Munich, Germany with a waiver of informed consent. The study protocol conformed to the ethical guidelines of the 1975 Declaration of Helsinki, as revised in 1983. All methods were performed in accordance with the relevant guidelines and regulations. We searched the institutional database of the Department of Neurosurgery at the Ludwig Maximilians University School of Medicine for adult patients with histologically verified supratentorial glioma WHO grade 2 seen between 2015 and 2019. Histopathologic diagnosis was based upon tissue sampled during microsurgical tumor removal, or stereotactic biopsy in lesions where safe resection appeared not feasible. In cases with and without tumoral contrast-enhancement on imaging, patients often received pre-operative brain tumor O-(2-[18F]-fluoroethyl)-L-tyrosine (FET) PET^14^. Stereotactic biopsy was eventually taken from contrast-enhancing foci and from hot-spots on PET imaging to avoid underdiagnosis. For patients in which biopsy and microsurgical tumor removal were both administered within 30 days after first presentation, only histopathology from tissue sampled during microsurgical tumor removal was reviewed. Tumors were classified according the 2016 WHO classification system. Diagnostic and treatment decisions were based upon interdisciplinary brain tumor board recommendations and patient preference. In patients with suspected tumor progression, stereotactic biopsy to establish diagnosis of tumor recurrence is typically provided at our institution. We collected demographic and clinical information, histopathology, radiographic and other diagnostic findings, treatment specifics, and clinical outcome. Database closure was September 1, 2019.

A subset of the included patients has partly been reported in a previous study on molecular markers in low-grade glioma^15^.

### Imaging review

Imaging characteristics were established through review of pre-treatment magnetic resonance imaging (MRI) of the brain. Patients without pre-treatment imaging were excluded. Particular attention was paid to subventricular zone involvement on T2-weighted imaging (defined as contact between tumor margins and lateral ventricle), maximal tumor diameter on T2-weighted imaging, and contrast-enhancement on post-contrast T1-weighted imaging. In patients with subventricular zone involvement, the anatomical subventricular subregion (frontal horn, body, occipital horn, temporal horn) involved by the tumor was recorded.

### Statistical analysis

Data were tested for normal distribution and equal variance using the D'Agostino-Pearson omnibus test. Differences between two groups were analysed by the unpaired Student’s t-test. In case of non-parametric data, differences between the groups were assessed by the Mann–Whitney U-test. All values are expressed as mean ± standard error of the mean if not indicated otherwise, and range is provided. Categorical variables are described in absolute numbers and percentage points. Relationships between categorical variables were analysed using Fisher's exact test. For survival analyses, patients were followed until death or day of database closure (September 1, 2019). Patients lost to follow-up were censored at the day of last follow-up. Date of diagnosis was set as the date of pathological glioma WHO grade 2 confirmation. Date of malignant progression was defined as the date when tissue-based diagnosis of malignant progression to WHO grade 3/4 was made, or death from any cause occurred. Overall survival was defined as interval from diagnosis to death from any cause. Follow-up, survival, and predictors of outcome were calculated using Kaplan–Meier survival analysis and log-rank test. Statistical analyses were performed using Prism statistical software (Prism 7.0a; GraphPad Software Inc., San Diego, CA, USA). The association of continuous variables or subventricular zone involvement and outcome was further assessed using Cox's proportional-hazard regression model to estimate hazard ratio (HR) and 95% confidence interval (CI). The one-in-ten rule was followed to adjust the number of parameters in multivariate analysis, and covariates which were available in > 90% of patients were included. Cox's proportional-hazard regression model was performed using SPSS statistical software (SPSS Statistics 26.0; IBM Corp., Armonk, NY, USA). The significance level was set at *p* ≤ 0.05.

## Results

### Study population

182 patients with supratentorial glioma WHO grade 2 were identified (Table 1). Patients were treated for the following underlying histopathologies: oligodendroglioma (97/182 patients, 53%); diffuse astrocytoma (79/182, 43%); gemistocytic astrocytoma (3/182, 2%); pleomorphic xanthoastrocytoma (2/182, 1%); and protoplasmic astrocytoma (1/182, 1%). 78 of 182 patients presented with glioma involving the subventricular zone at time of initial diagnosis, and the relative incidence of subventricular zone involvement was therefore estimated to be 43%. Median age in patients with subventricular zone involvement was 40 ± 1.7 years (range 20–81 years) and 38 ± 1.1 years (range 18–66 years) in patients without subventricular zone involvement (*p* = 0.283). Male-to-female ratio was comparable between patients with subventricular zone involvement (1:1.3) and patients without subventricular zone involvement (1:0.9) (*p* = 0.296).

**Table 1:** Patient characteristics for WHO grade 2 gliomas with and without involvement of the subventricular zone.

Glioma localization		Subventricular	Non-subventricular	Total	*p* value
Overall, n (%)		78 (43%)	104 (57%)	182	
**Age, years**	18–35	31 (40%)	36 (35%)	67 (37%)	0.283
	36–50	26 (33%)	50 (48%)	76 (42%)	
	51–65	12 (15%)	15 (14%)	27 (15%)	
	> 65	9 (12%)	3 (3%)	12 (7%)	
**Gender**	Female	44 (56%)	50 (48%)	94 (52%)	0.296
	Male	34 (44%)	54 (52%)	88 (48%)	
**KPS**	< 90	13 (17%)	5 (5%)	18 (10%)	0.003*
	90–100	45 (58%)	81 (78%)	126 (69%)	
	n.a.	20 (26%)	18 (17%)	38 (21%)	
**Symptoms**	Seizures	47 (60%)	54 (52%)	101 (56%)	
	No symptoms	1 (1%)	6 (6%)	7 (4%)	
**Histopathology**	ODG	39 (50%)	58 (56%)	97 (53%)	
	Diffuse AST	35 (45%)	44 (42%)	79 (43%)	
	Gemistocytic AST	3 (4%)	0	3 (2%)	
	PP AST	1 (1%)	0	1 (1%)	
	PXA	0	2 (2%)	2 (1%)	
**Molecular markers**	IDH mutation	62 (80%)	94 (90%)	156 (86%)	0.107
	IDH wildtype	13 (17%)	9 (9%)	22 (12%)	
	IDH status n.a.	3 (4%)	1 (1%)	4 (2%)	
	1p19q codeletion	39 (50%)	58 (56%)	97 (53%)	0.453
	No 1p19q codeletion	39 (50%)	45 (43%)	84 (46%)	
	1p19q status n.a.	0	1 (1%)	1 (1%)	
	MGMT methylated	70 (90%)	99 (95%)	169 (93%)	0.059
	MGMT unmethylated	8 (10%)	3 (3%)	11 (6%)	
	MGMT status n.a.	0	2 (2%)	2 (1%)	
	TERT mutation	29 (37%)	37 (36%)	66 (36%)	0.846
	TERT wildtype	18 (23%)	25 (24%)	43 (24%)	
	TERT status n.a.	31 (40%)	42 (40%)	73 (40%)	
**Tumor diameter**	0–2.5 cm	3 (4%)	20 (19%)	23 (13%)	0.001*
	2.6–5 cm	14 (18%)	58 (56%)	72 (40%)	
	5.1–7.5 cm	40 (51%)	20 (19%)	60 (33%)	
	> 7.5 cm	20 (26%)	1 (1%)	21 (12%)	
	n.a.	1 (1%)	5 (5%)	6 (3%)	
**First-line therapy**	Surgical resection				0.868
	GTR	9 (12%)	20 (19%)	29 (16%)	
	STR	13 (17%)	8 (8%)	21 (12%)	
	Chemotherapy				0.004*
	TMZ	28 (36%)	18 (17%)	46 (25%)	
	PCV	0	4 (4%)	4 (2%)	
	PC	15 (19%)	13 (13%)	28 (15%)	
	Radiotherapy	10 (13%)	21 (20%)	31 (17%)	0.234
	Radiochemotherapy	6 (8%)	13 (13%)	19 (10%)	0.336
	Brachytherapy	1 (1%)	14 (14%)	15 (8%)	0.002*
	Watch-and-scan	14 (18%)	30 (29%)	44 (24%)	0.115

Characteristics are given for WHO grade 2 glioma patients with involvement of the subventricular zone (subventricular; n = 78), without involvement of the subventricular zone (non-subventricular; n = 104), and are summarized for all patients (total; n = 182). *p* values are given for numerical and dichotomous variables. Asterisks indicate *p* ≤ 0.05.

*AST* astrocytoma, *GTR* gross total resection, *IDH* isocitrate dehydrogenase 1/2, *KPS* Karnofsky performance score, *MGMT* O6-methylguanine-DNA methyltransferase promotor, *n.a.* not available for review, *ODG* oligodendroglioma, *PC* procarbazine, lomustine, *PCV* procarbazine, lomustine, vincristine, *PP* protoplasmic, *PXA* pleomorphic xanthoastrocytoma, *STR* subtotal resection, *TERT* telomerase reverse transcriptase promotor, *TMZ* temozolomide.

Among all 182 patients included in our study, 156 patients had an isocitrate dehydrogenase (IDH) mutation (86%), 1p19q codeletion was found in 97 patients (53%), O6-methylguanine-DNA methyltransferase promotor (MGMT) methylation was seen in 169 patients (93%), and 66 patients had a telomerase reverse transcriptase promotor (TERT) mutation (36%) (Table 1). There were no differences between patients with or without subventricular zone involvement in regards to IDH mutation status, 1p19q codeletion status, MGMT methylation status, and TERT mutation status. When patients were stratified according to the recent cIMPACT-NOW update 6^12^, we encountered 65 IDH-mutant astrocytomas (including the gemistocytic and protoplasmic astrocytomas; 26 with subventricular zone involvement, 39 without subventricular zone involvement), 97 1p19q-codeleted oligodendrogliomas (39 with subventricular zone involvement, 58 without subventricular zone involvement), and 20 IDH-wildtype astrocytomas (including the pleomorphic xanthoastrocytomas; 13 with subventricular zone involvement, 7 without subventricular zone involvement).

### Clinical and radiographic findings

The majority of patients were symptomatic at time of diagnosis, and the most frequently reported symptoms were attributed to tumor mass effect: generalized or focal seizures (47/78 patients with subventricular zone involvement, 60%; 54/104 patients without subventricular zone involvement, 52%); headache (21/78, 27%; 20/104, 19%); confusion or neuropsychological deficits (20/78, 26%; 9/104, 9%); and vertigo or gait instability (8/78, 10%; 11/104, 11%). Seven gliomas, including one tumor with subventricular zone involvement (1/78, 1%) and six tumors without subventricular zone involvement (6/104, 6%), were discovered incidentally. Karnofsky performance score differed between patients with and without subventricular zone involvement: significantly more patients with Karnofsky performance score lower 90 were encountered among individuals with subventricular zone involvement (13/78, 17%) when compared to individuals without subventricular zone involvement (5/104, 5%) (*p* = 0.003).

**Figure 1:**
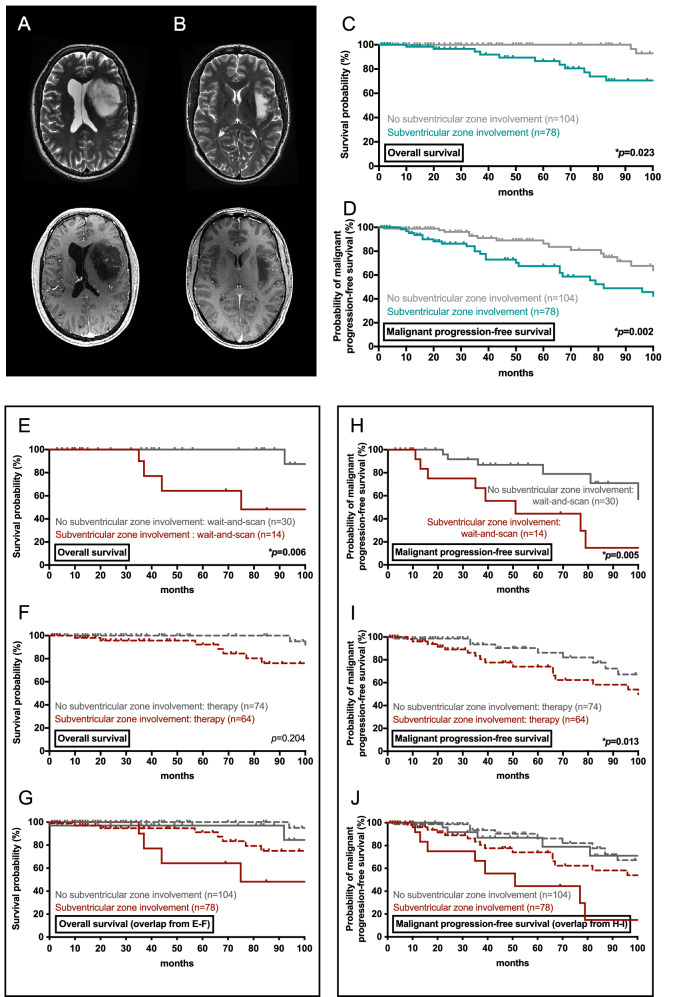
Subventricular zone involvement as prognostic marker in glioma WHO grade 2. (**A**) Axial brain MRI with T2-weighted (upper panel) and T1-weighted post-contrast (lower panel) sequences shows diffuse astrocytoma with small foci of contrast-enhancement and subventricular zone involvement (**A**; left) and diffuse astrocytoma without contrast-enhancement or subventricular zone involvement (**B**; right). (**C**, **D**) Kaplan–Meier estimates of overall survival (**C**) and malignant progression-free survival (**D**) for the entire cohort of glioma WHO grade 2 patients with and without subventricular zone involvement. (**E**–**J**) Kaplan–Meier estimates of overall survival (**E**–**G**) and malignant progression-free survival (**H**–**J**) for different patient subgroups with and without subventricular zone involvement. Curves are given for the subgroup of patients which were managed with a wait-and-scan approach (**E**, **H**) and patients which were managed with therapy other than wait-and-scan (**F**, **I**). (**G**) (and **J**) represents an overlap of the curves from (**E**, **F**) (and **H**, **I**) to visualize the difference in outcome between patients who received wait-and-scan approaches (straight lines) and patients who received first-line therapy (dotted lines) as stratified according to subventricular zone involvement (patients with subventricular zone involvement: red lines; patients without subventricular zone involvement: grey lines). Tick marks indicate censored patients.

Pre-treatment brain MRI studies showed intra-axial hyperintense lesions with ill-defined margins on T2-weighted sequences in all patients (Fig. 1A,B). Among the 78 patients with subventricular zone involvement, lesions often contacted more than one subregion of the subventricular zone. Tumors involved the frontal horn of the lateral ventricle in 44 patients (56%), the ventricle body in 33 patients (42%), the temporal horn in 25 patients (32%), and the occipital horn in 22 patients (28%). Maximal tumor diameter was measured on T2-weighted sequences, and diameters were significantly higher at time of diagnosis in individuals with subventricular zone involvement when compared to patients without subventricular zone involvement (6.2 ± 0.2 vs. 3.9 ± 0.1 cm; *p* = 0.001). Contrast-enhanced T1-weighted studies were available in 75 of 78 patients with subventricular zone involvement, and in 95 of 104 patients without subventricular zone involvement. Whereas vivid or patchy contrast-enhancement was seen in 35 of 75 patients with subventricular zone involvement (47%), only 13 of 95 patients without subventricular zone involvement (13%) developed intra-tumoral contrast-enhancing foci (*p* = 0.001). Of note, surgical tumor resection was administered in 10 of 35 patients with contrast-enhancing tumors and subventricular zone involvement (29%), and in 4 of 13 patients with contrast-enhancing tumors without subventricular zone involvement (31%). Diagnosis rested upon stereotactic biopsy from contrast-enhancing foci in the remaining cases, and pathology was consistent with glioma WHO grade 2 in all patients despite contrast-enhancement on MRI. In patients without contrast-enhancement on MRI and who underwent biopsy only to establish tissue-based diagnosis (n = 105), brain tumor PET was often performed to identify the most active and diagnostically relevant biopsy target (50/105 patients, 48%) but was not deemed necessary when a clear solid tumor focus was identified on conventional imaging (45/105 patients, 43%; data not available in 10/105 patients, 10%)^16^.

Among patients with subventricular zone involvement, mean distance from the tumor centroid to the subventricular zone was 0.78 ± 0.2 cm in IDH-wildtype astrocytomas, 1.80 ± 0.2 cm in IDH-mutant astrocytomas, and 1.92 ± 0.2 cm in 1p19q-codeleted oligodendrogliomas. Therefore, the mean distance was significantly lower in IDH-wildtype astrocytomas compared to IDH-mutant astrocytomas (*p* = 0.002) and 1p19q-codeleted oligodendrogliomas (*p* = 0.001), whereas there was no difference between IDH-mutant astrocytomas and 1p19q-codeleted oligodendrogliomas (*p* = 0.586). Notably, the tumor diameter of 1p19q-codeleted tumors was also larger than the diameter of IDH-wildtype astrocytomas (7.1 ± 0.5 cm vs. 5.1 ± 0.6 cm, *p* = 0.016) and IDH-mutant astrocytomas (7.1 ± 0.5 cm vs. 5.8 ± 0.3 cm, *p* = 0.038). The difference in tumor diameter between IDH-wildtype astrocytomas and IDH-mutant astrocytomas was not of significance (*p* = 0.282).

### Treatment and outcome

First-line management of glioma WHO grade 2 included surgical tumor resection, systemic chemotherapy (predominantly temozolomide or procarbazine/lomustine), involved-field radiotherapy, interstitial brachytherapy (for small, not safely resectable lesions), and wait-and-scan approaches (Table 1). Patients with subventricular zone involvement received more frequently chemotherapy when compared to patients without subventricular zone involvement, whereas more patients without subventricular zone involvement received brachytherapy as first-line approach.

Median follow-up time was 43 months (range 0–330 months). Median time to malignant progression was 122 months; median overall survival was not reached after 200 months. 47 patients (26%) had malignant tumor progression as determined by surgical tumor resection or biopsy (four patients within the first twelve months after initial diagnosis), and eighteen patients (10%) were deceased at time of data cutoff (one patient within the first twelve months after initial diagnosis), including twelve patients with subventricular zone involvement and six patients without subventricular zone involvement. Among those who suffered malignant tumor progression, median time to malignant progression was 60 months (range 2–178 months). There was no difference in regards to the therapy provided after tumor progression between patients with and without subventricular zone involvement.

### Prognostic markers

In the entire cohort, presentation with subventricular zone involvement (n = 78) was a significant negative prognostic marker for overall survival (*p* = 0.023) and time to malignant progression (*p* = 0.002) when compared to patients without subventricular zone involvement (n = 104) on univariate analysis (Fig. 1C,D). Given the exceedingly rare number of events in the first 12 months after diagnosis, this also held true when only patients with a follow-up time of ≥ 12 months (n = 149) were included in the outcome analysis. On multivariate analysis, the significant association of subventricular zone involvement and worse outcome was further confirmed (Table 2). The subventricular zone involvement was associated with inferior overall survival (HR 3.73, CI 1.1–12.7) and a shorter time to malignant progression (HR 2.81, CI 1.4–5.5) when tested together with demographic, molecular, and therapeutic covariates. When patients were stratified according to the cIMPACT-NOW update 6, subventricular zone involvement was negatively associated with outcome in patients with 1p19q-codeleted oligodendrogliomas (overall survival: *p* = 0.007; malignant progression: *p* = 0.103) and IDH-wildtype astrocytomas (overall survival: *p* = 0.077; malignant progression: *p* = 0.037), but not in patients with IDH-mutant astrocytomas (overall survival: *p* = 0.813; malignant progression: *p* = 0.537) (using Kaplan–Meier survival analysis and log-rank test; Supplementary Figure 1). Notably, this also held true when patients with gemistocytic and protoplasmic astrocytomas were excluded from the group of IDH-mutant astrocytomas, and patients with pleomorphic xanthoastrocytomas were excluded from the group of IDH-wildtype astrocytomas. Closer proximity of the tumor centroid to the subventricular zone was not associated with outcome in IDH-wildtype astrocytomas (distance < 0.78 cm vs. ≥ 0.78 cm; overall survival: *p* = 0.502, malignant progression: *p* = 0.277), IDH-mutant astrocytomas (distance < 1.80 cm vs. ≥ 1.80 cm; overall survival: *p* = 0.578, malignant progression: *p* = 0.566), and 1p19q-codeleted oligodendrogliomas (distance < 1.92 cm vs. ≥ 1.92 cm; overall survival: *p* = 0.655, malignant progression: *p* = 0.162) with subventricular zone involvement.

**Table 2:** Multivariate Cox's overall survival and malignant progression-free survival analysis.

Covariate	Overall survival			Malignant progression-free survival		
	Hazard ratio	95% confidence interval	*p* value	Hazard ratio	95% confidence interval	*p* value
Subventricular zone involvement	3.73	1.1–12.7	0.036*	2.81	1.4–5.5	0.003*
**Molecular markers**						
IDH mutation	0.92	0.0–0.5	0.006*	0.33	0.1–1.0	0.049*
MGMT methylation	0.22	0.0–1.4	0.111	0.38	0.1–1.1	0.082
1p19q codeletion	0.47	0.1–1.6	0.236	0.52	0.3–1.0	0.061
**Demographics**						
Age	1.01	1.0–1.1	0.784	0.99	1.0–1.0	0.430
Male sex	2.35	0.8–7.0	0.125	1.52	0.8–2.9	0.212
**Therapy**						
Surgical resection	0.89	0.2–4.8	0.896	0.94	0.3–2.9	0.915
Medical therapy	1.82	0.3–11.1	0.518	1.00	0.3–3.2	1.000
Wait-and-scan	1.33	0.2–11.5	0.798	1.17	0.3–4.6	0.819

Multivariate overall survival and malignant progression-free survival analysis was performed among WHO grade 2 glioma patients (n = 182) using clinical and molecular covariates. Molecular markers, sex, and therapeutic approaches were tested as dichotomous variables, and age was tested as continuous variable. Hazard ratio, 95% confidence interval, and *p* value are given for analyzed covariates. Asterisks indicate *p* ≤ 0.05.

*IDH* isocitrate dehydrogenase 1/2, *MGMT* O6-methylguanine-DNA methyltransferase promotor.

We aimed to analyse whether subventricular zone involvement represents an independent risk factor when accounting for the extent of resection and the respective tumor size. In the subgroup of patients which did not undergo surgical tumor resection but received a biopsy only (n = 132), subventricular zone involvement was still significantly associated with less favourable outcome (Table 3). This was particularly evident for tumors ≤ 5 cm when patients with and without subventricular zone involvement were stratified for tumor size, and no differences for IDH mutation were seen in such patients. We further assessed whether the differences in outcome were due to differences in performance score, tumor size, appearance on imaging, or age. Of interest, Karnofsky performance score (tested as a continuous variable), tumor diameter (tested as a continuous variable), and contrast-enhancement on MRI (n = 48) appeared to be neither associated with overall survival (Karnofsky performance score: *p* = 0.286; tumor size: *p* = 0.859; contrast-enhancement: *p* = 0.785) nor with time to malignant progression (Karnofsky performance score: *p* = 0.920; tumor size: *p* = 0.247; contrast-enhancement: *p* = 0.267). Age tested as a continuous variable was a marker for short overall survival (*p* = 0.016) but not malignant progression (*p* = 0.971).

**Table 3:** Subventricular zone involvement as prognostic marker in glioma WHO grade 2 with biopsy only.

Tumor diameter	Number of patients		IDH mutations (%)			Malignant progression-free survival			Overall survival		
	SV	Non-SV	SV	Non-SV	*p* value	Hazard ratio	95% confidence interval	*p* value	Hazard ratio	95% confidence interval	*p* value
≤ 2.5 cm	3	14	1 (33%)	13 (93%)	0.063	0.01	0.00–0.01	0.001*	0.01	0.00–0.25	0.014*
2.6–5 cm	12	44	9 (75%)	37 (84%)	0.433	0.01	0.00–0.01	0.001*	0.08	0.01–0.74	0.026*
≥ 5.1 cm	41	17	31 (76%)	17 (100%)	0.026*	0.30	0.11–0.86	0.025*	0.38	0.08–1.98	0.249
n.a.	0	1	0	1 (100%)	–	–	–	–	–	–	–

Univariate malignant progression-free and overall survival analysis for subventricular zone involvement as prognostic marker was performed among WHO grade 2 glioma patients (n = 132), which did not undergo surgical tumor resection but biopsy only. Patients were stratified for tumor size, and IDH mutation status is indicated. Hazard ratio, 95% confidence interval of hazard ratio, and *p* value are given. Outcome analyses (malignant progression-free survival, overall survival) were performed using Kaplan–Meier survival analysis and log-rank test. Asterisks indicate *p* ≤ 0.05.

*IDH* isocitrate dehydrogenase 1/2, *n.a.* not available for review, *non-SV* non-subventricular, *SV* subventricular, *–*undefined.

We aimed to identify whether the effect of subventricular zone involvement on outcome holds true when tested in different treatment-based subgroups. Within the subgroup of patients in which only a first-line wait-and-scan surveillance strategy was provided, subventricular zone involvement (n = 14) was a predictive marker for both worse overall survival (*p* = 0.006) and shorter time to malignant progression (*p* = 0.005) (Fig. 1E,H); however, within the subgroup of patients in which early therapy (surgical tumor resection, chemotherapy, radiotherapy, or brachytherapy) was administered, subventricular zone involvement (n = 64) was not a negative prognostic marker for overall survival (*p* = 0.204) (Fig. 1F,I). Among patients who received early therapy, 5-year survival rates were 100% for patients without subventricular zone involvement and 92% for patients with subventricular zone involvement. The effect of early therapy seemed to diminish over time, and 7-year survival rates were 100% for patients without subventricular zone involvement and 76% for patients with subventricular zone involvement (Fig. 1G,J). Of note, the use of first-line resection (including extent of resection in particular), chemotherapy, radiotherapy, radiochemotherapy, or brachytherapy were all not associated with outcome (when tested against patients who did not receive the respective treatment).

Among the subgroup of patients with subventricular zone involvement, we found that IDH mutation (n = 62) and MGMT methylation (n = 70) were also positive prognostic indicators for overall survival (IDH mutation: *p* = 0.001; MGMT methylation: *p* = 0.001) and time to malignant progression (IDH mutation: *p* = 0.001; MGMT methylation: *p* = 0.001). Involvement of the subventricular zone bordering the frontal horn of the lateral ventricle (n = 44) did not bear a distinct prognostic effect on overall survival (*p* = 0.659) or time to malignant progression (*p* = 0.385) when compared to patients with involvement of other subventricular subregions.

## Discussion

The subventricular zone is considered to harbour the highest number of neural stem cells in the adult brain^6^. The impact of subventricular zone involvement on the biology and clinical course of glioma WHO grade 2 is not well understood, but might be of relevance in regards to management and outcome. Based on a large cohort of 182 patients, we here present the institutional experience for incidence, management, and outcome of subventricular zone involvement in such tumors.

We found an incidence of 43% for subventricular zone involvement among 182 patients with glioma WHO grade 2. Subventricular zone involvement was associated with decreased overall survival and shorter time to malignant progression in the entire cohort. By the date of data cutoff, twelve of 78 patients with subventricular zone involvement had died, whereas only six of 104 patients without subventricular zone involvement were deceased. This observation corroborates the hypothesis that subventricular zone involvement constitutes a risk factor for worse outcome. Of note, we did not find evidence that this finding was due to other risk factors such as age, gender, or use of tumor resection. Although performance score and frequency of chemotherapy as well as brachytherapy provided as first-line therapy differed between patients with and without subventricular zone involvement, these factors were not prognostic in our cohort. Importantly, subventricular zone involvement was still associated with less favourable outcome among patients who did not receive microsurgical tumor resection, and when patients were stratified for tumor size. Thus, differences in the extent of resection or tumor size between patients with and without subventricular zone involvement likely did not confound our analysis.

When patients were stratified based on molecular markers according to the cIMPACT-NOW update 6^12^, subventricular zone involvement was a risk factor in IDH-wildtype astrocytomas and 1p19q-codeleted oligodendrogliomas but not in IDH-mutant astrocytomas. This important finding may reflect inherent biological differences between these tumor entities formerly summarized under WHO grade 2. Previous studies have provided compelling evidence that neural stem cells within the subventricular zone drive growth and recurrence of high-grade glioma^7,17^. We cannot comment on whether similar biological mechanisms may have played a role in malignant progression and tumor recurrence within our cohort; however, this hypothesis needs to be considered in future studies. Notably, previous studies on diffuse astrocytomas WHO grade 2, which have reported that close proximity to the subventricular zone is correlated with shorter long-term survival, did not adequately control for IDH- or 1p19q-codeletion-status^10,11,18^. In tumors with subventricular zone involvement, we did not find that closer proximity to the subventricular zone was associated with distinct outcome. However, we found that IDH-wildtype astrocytomas with subventricular zone involvement were closest to the subventricular zone compared to IDH-mutant astrocytomas and 1p19q-codeleted tumors. This may reflect the somewhat smaller diameters in such tumors, but also a potential interaction of IDH-wildtype astrocytomas and the subventricular zone in tumor formation. This would be in line with the recent assumption that IDH-wildtype astrocytomas molecularly reassemble glioblastomas^13^, and that a subset of glioblastomas origins from the subventricular zone^7^. Of note, the therapeutic differences between patients with and without subventricular zone involvement (also within molecular subgroups) may represent a potential confounder of our study. Although validation of our results in prospective, homogenously treated cohorts is therefore warranted, our findings suggest that these studies will urgently need to stratify patients according to their molecular signature.

Our analysis showed that the negative effect of subventricular zone involvement on outcome was ameliorated in patients receiving early therapy. When managed with therapeutic approaches other than wait-and-scan, no difference in overall survival was found between patients with and without subventricular zone involvement. Our data may therefore suggest that early therapy might form the basis for favourable outcome in glioma WHO grade 2 with subventricular zone involvement. Accordingly, wait-and-scan approaches should be applied with caution in such patients. If this assumption would hold true when evaluated in prospective clinical trials is unclear, and no definitive treatment recommendation can be made at this point based on the findings from our study. Particularly, molecular markers will need to be considered when making treatment decisions^19^. However, prior prospective studies have demonstrated that if overall survival is prioritized, radiochemotherapy might be recommended in patients with glioma WHO grade 2^2,19,20^. We did not find a significant association of use of radiochemotherapy and outcome in our cohort, potentially due to the small sample size of only 19 patients treated with radiochemotherapy. Moreover, these 19 patients had considerable risk factors for worse outcome including larger tumor volumes and contrast enhancing on imaging, which may have ameliorated the beneficial effects of radiochemotherapy.

In our present study, the survival benefit from early therapy among patients with subventricular zone involvement seemed to diminish over time. It has previously been shown that the effect of early therapy is particularly pronounced within the initial period after treatment of glioma WHO grade 2^4^. However, more recent studies by others have demonstrated that glioma WHO grade 2 patients with adverse prognostic markers may particularly benefit from early therapy^3,21,22^. There was no effect of early therapy on the excellent outcome of patients without subventricular zone involvement in our cohort. On a cautionary note, we encountered a relatively low number of patients who received surgical tumor resection as first-line therapy. This might be due to the fact that many patients seen at our institution seem to prefer medical therapy over early resection, and shared decision was made. Long-term toxicities have to be taken into consideration when administering therapy given the relatively long survival of patients with gliomas WHO grade 2^23^. Brachytherapy is considered to deliver radiation dose to a well-defined tumor target^24^, whereas involved-field radiotherapy in glioma patients often includes irradiation of adjacent parenchymal tissue. Retrospective studies by others have shown that administering higher radiation doses to the subventricular zone may prolong survival^25^. Whether the subventricular zone represents a potential therapeutic target in such patients is being investigated and remains elusive^25^. The effects of such therapy will need to be carefully assessed for long-term cognitive outcome given the importance of the subventricular zone for brain plasticity^26^.

Specific points and limitations of the study are a large cohort with a considerable follow-up time, however, we encountered only a small number of patients meeting the endpoints death and tumor progression. Also, we included patients with a short follow-up time of less than 12 months in our cohort to analyse demographic and molecular markers of subventricular zone involvement. However, all of our key findings held true when re-calculated only including patients with a follow-up time ≥ 12 months. Although treatment in our cohort was based on 'standard of care' at time of the patients' diagnosis, this treatment might not be representative for a number of patients newly diagnosed with glioma WHO grade 2 in 2021^2^. Based on these limitations, our findings on the prognostic role and therapeutic implications of subventricular zone involvement in glioma WHO grade 2 warrant evaluation in prospective cohorts which consider novel prognostic markers such as CDKN2A/2B deletion.

Our review of pre-treatment imaging showed that gliomas WHO grade 2 with subventricular zone involvement presented with larger size and were more often accompanied by contrast-enhancing foci than gliomas without subventricular zone involvement. Seizures were the most commonly reported symptom in both groups at initial diagnosis. We therefore hypothesize that larger tumor size might be due to the fact that deep-seated tumors may be asymptomatic for a longer time given the anatomical distance to cerebral cortical structures. The role of contrast-enhancement in the outcome of glioma WHO grade 2 is discussed controversially^27^, but close follow-up should be advised in patients with contrast-enhancing foci to identify progression^28^. Contrast-enhancement on MRI was not associated with worse outcome in our cohort, and diagnosis of glioma WHO grade 2 rested upon tissue sampled during surgical tumor removal in many patients with contrast-enhancing foci. In the remaining patients with contrast-enhancement that were managed with stereotactic biopsy, tissue was particularly sampled from the contrast-enhancing area to reduce sample bias and underdiagnosis. However, we cannot fully rule out sampling bias in such patients. Advanced imaging modalities such as brain tumor PET may be useful diagnostic tools to visualize the extent of disease or tumor recurrence in cases of diagnostic uncertainty^16^. Stereotactic tissue biopsy should be administered when malignant progression is suspected and needs to be distinguished from radiation necrosis or pseudoprogression^16,29^.

The frontal horn of the lateral ventricle was the most frequently involved subregion of the subventricular zone, which may reflect the large size of the frontal lobe. The anterior subventricular zone is considered to harbour a particularly high cellular turnover^30^. However, we did not find evidence that involvement of the frontal subventricular zone confers an elevated risk for progression or worse outcome.

In conclusion, subventricular zone involvement may represent a key risk factor for worse outcome in glioma WHO grade 2 depending on the individual molecular tumor signature. Early therapy may form the basis for more favourable outcomes in such patients, and wait-and-scan approaches should be used with caution. Our findings warrant evaluation in molecularly well-defined, homogenously treated prospective cohorts. Understanding the biological role of the subventricular zone in tumorigenesis of glioma WHO grade 2 and identifying molecular factors that serve as potential therapeutic targets may represent a promising approach in future management of these patients.

## Supplementary Information

Supplementary Information.

## Data Availability

All relevant data are within the paper. All data were kept anonymous and are available on qualified request.
