# Supplementary material for: Subventricular zone involvement is associated with worse outcome in glioma WHO grade 2 depending on molecular markers
Source: Sci Rep. 2021 Oct 8;11:20045. doi: 10.1038/s41598-021-97714-5 (PMC8501091; doi:10.1038/s41598-021-97714-5)
Supplement: Supplementary file 1 — Supplementary Information. [file 41598_2021_97714_MOESM1_ESM.pdf]

Supplementary Figure 1

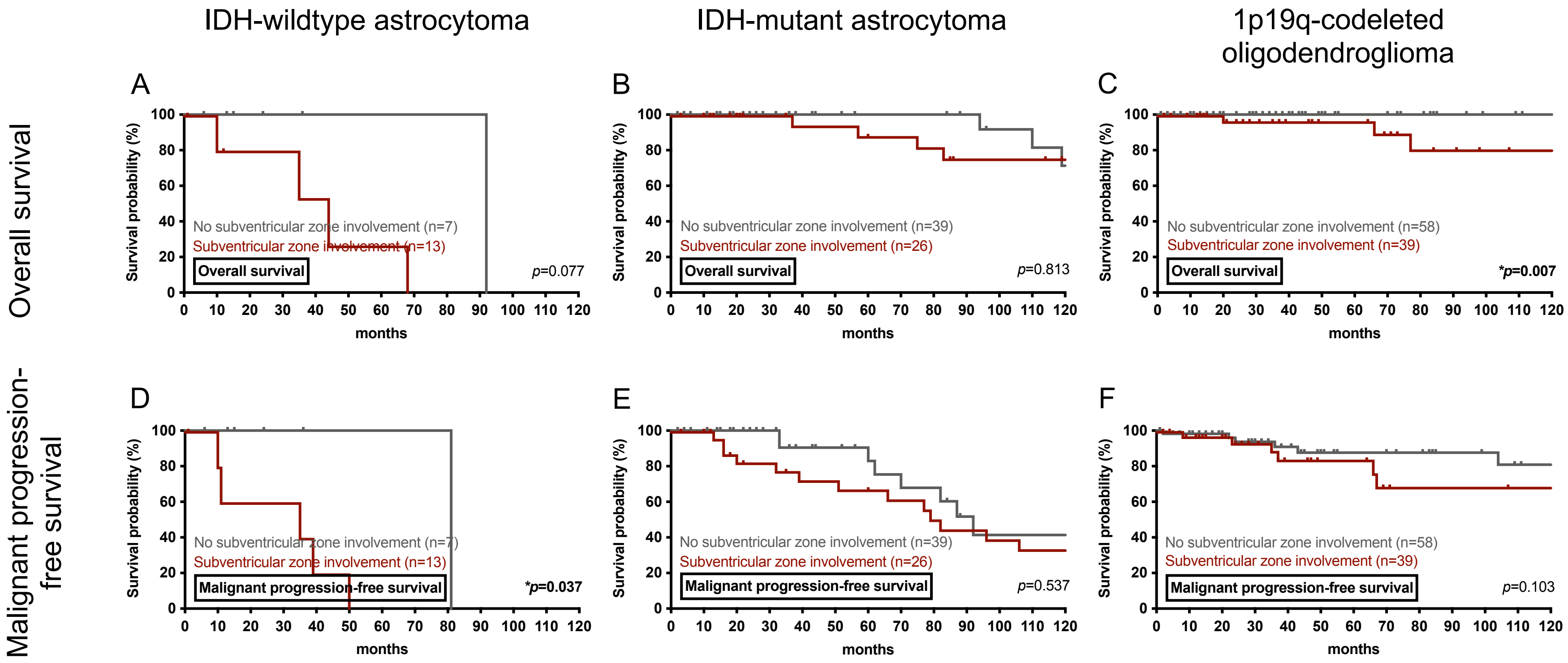

Supplementary Figure 1: Patients with and without subventricular zone involvement stratified according to the cIMPACT-NOW update 6. Kaplan-Meier estimates of overall survival (A-C) and malignant progression-free survival (D-F) in patients with IDH-wildtype astrocytomas (A, D; n = 20), IDH-mutant astrocytomas (B, E; n = 65), and 1p19q-codeleted oligodendrogliomas (C, F; n = 97). Curves are given for patients with subventricular zone involvement (red lines) and patients without subventricular zone involvement (grey lines). Tick marks indicate censored patients.
